# The challenge of diagnosing intracranial pressure elevations as an otolaryngologist

**DOI:** 10.1007/s00405-025-09333-9

**Published:** 2025-03-28

**Authors:** Michelle S. Klausner, Gerard J. Gianoli, Patricia Johnson, Bulent Mamikoglu

**Affiliations:** 1 New York Medical College School of Medicine, Valhalla, NY United States of America; 2 The Ear and Balance Institute, 1401 Ochsner Blvd. Suite A, Covington, LA United States of America; 3 Department of Otolaryngology-Head and Neck Surgery, Tulane University School of Medicine, New Orleans, LA United States of America; 4 Department of Otolaryngology, Westchester Medical Center, Valhalla, NY United States of America

**Keywords:** Migraine, Headache, Chronic rhinosinusitis, Mid-facial pain, Tension headache, Elevated intracranial pressure

## Abstract

**Purpose:**

This article addresses the complex clinical scenario where patients present to otolaryngologists with symptoms typically ascribed to chronic rhinosinusitis (CRS) or migraines which may in fact stem from elevations in intracranial pressure. We aim to clarify the diagnostic challenges and emphasize the importance of considering elevated intracranial pressure (eICP) as its symptoms overlap with both CRS and migraines.

**Methods:**

This narrative review synthesizes clinical experiences and literature to discuss the differential diagnoses involving CRS, facial pain/pressure, migraines, and eICP. Key discussion points include symptomatology of eICP and its management in otolaryngological practice.

**Results:**

Patients presenting with symptoms of CRS or migraine may exhibit overlapping signs that makes diagnosis challenging. Patients with symptoms of facial pain and pressure, or other findings such as ear fullness, muffled hearing, and tinnitus, that do not resolve with conventional topical intranasal therapies or migraine management should be worked up for eICP.

**Conclusion:**

The overlap in clinical presentations among patients with concern for CRS, migraines, and ICP elevations poses a diagnostic challenge. It is crucial for otolaryngologists and neurologists to collaborate closely to ensure accurate diagnoses and appropriate management. Enhanced awareness and understanding of the broader spectrum of symptoms associated with eICP can prevent misdiagnosis and promote better patient outcomes.

## Introduction

A frequent presentation for otolaryngologists is patients with facial pain or pressure or ear pressure without evidence of sinusitis or eustachian tube dysfunction. While these patients are often diagnosed with migraine or chronic rhinosinusitis (CRS), elevated Intracranial Pressure (eICP) can produce an identical presentation, particularly in the absence of papilledema.

One explanation for the overlap in symptoms between migraines and eICP is the overlap in risk factors. While the causes for migraines are multifactorial, several modifiable and non-modifiable risk factors exist. Non-modifiable risk factors include age, female sex, and previous head trauma, while modifiable risk factors include medication overuse, obesity, and stressful life events [1]. Similarities in risk factors are shared with IIH, which is associated with obesity, female sex, and younger adults. Thus, a proportion of patients who suffer from migraines have a concurrent cerebrospinal fluid (CSF)-related issue like idiopathic intracranial hypertension (IIH) [2–5]. While risk factors for CRS generally differ from the risk factors listed above, the overlap in symptomatology between CRS and migraines renders arriving at a specific diagnosis challenging at times.

**Fig. 1:**
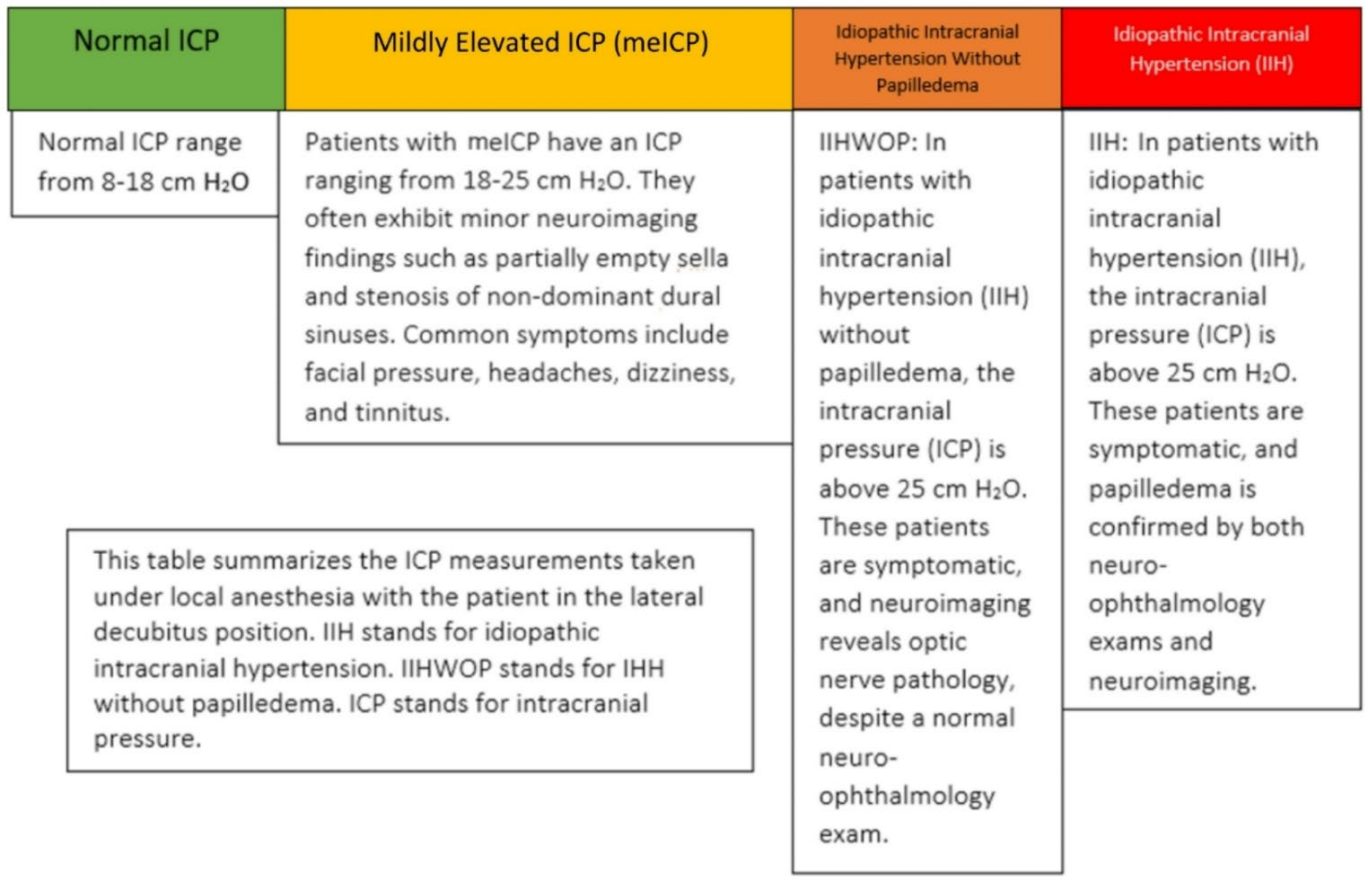
This figure outlines key differences among conditions associated with elevated intracranial pressure, with a focus on idiopathic intracranial hypertension (IIH). IIH is diagnosed when papilledema develops in the absence of an intracranialmass or tumor, due to elevated intracranial pressure (ICP). In some cases, IIH may occur without papilledema (IIHWOP), although strict imaging criteriaare required to confirm this diagnosis. Mildly elevated intracranial pressure (meICP) differs from both IIH and IIHWOP. These patients typically present with facial pressure, headache, and dizziness, but do not exhibit papilledema. Although their ICP is consistently elevated, it does not reach the threshold for intracranial hypertension

IIH is a condition characterized by raised ICP that is not due to a mass lesion or another known cause [6, 7]. It is diagnosed using the Friedman criteria: (1)papilledema, (2)normal neurologic exam except for cranial nerve abnormalities, (3)neuroimaging demonstrating no evidence of hydrocephalus, meningeal enhancement, or mass lesion, (4)normal CSF composition, and (5)elevated opening pressure > 25 cm H2O CSF at lumbar puncture [8]. A definitive diagnosis is made if all five criteria are met, and a likely diagnosis is made if criteria 1–4 are met [8]. A study by Sina et al. demonstrated that among 68 patients with known IIH, 45 (63.2%) met diagnostic criteria for migraine headaches, demonstrating the overlap that exists between the two disorders [2]. While facial pain is not the primary symptom of IIH, several cases have demonstrated that it may be the main or presenting symptom in patients experiencing eICP [9, 10]. In addition to IIH, other subgroups of eICP exist, with one pertinent subgroup being IIH without papilledema (IIHWOP), in which all of the Friedman criteria except papilledema are met, and patients additionally experience either unilateral or bilateral abducens nerve palsy or three imaging findings of: empty sella, flattening of the posterior aspect of the globe, distention of the perioptic subarachnoid space, or transverse sinus stenosis [8]. Another subgroup includes patients with mildly elevated ICP (meICP), but without papilledema. Patients falling into this category typically experience facial pressure, dizziness, and often have a history of migraines but do not fulfill the criteria for IIH or IIHWOP [11]. Furthermore, patients with meICP may have an ICP of 18–25 cm H2O (unlike the Friedman criteria, which requires ICP > 25) but demonstrate resolution of symptoms by lowering the ICP. Any of these circumstances may bring patients into the otolaryngologist’s office, necessitating understanding the various presentations of eICP (Fig. 1).

This article emphasizes the potential for headache and facial pain symptoms to be manifestations of eICP rather than primary sinus or migraine disorders. Here we explore novel updates in the understanding of eICP and IIH and the implications of headache diagnosis and management concerning the intersection of otolaryngology and neurology, as highlighted through three patient cases.

## Methods

This narrative review synthesizes clinical experiences and literature to discuss the differential diagnosis involving CRS, facial pain, migraines, and eICP. We reviewed a Medline and Pubmed search of peer-reviewed articles from 1994 through April 2024 with the key words including “idiopathic intracranial hypertension,” “pseudotumor cerebri,” “elevated intracranial pressure,” “migraine,” “sinus headache,” “chronic rhinosinusitis,” and “glymphatic system.”

### Etiology of eICP

CSF secretion, composition, and turnover are tightly regulated. A main theory underlying the cause of IIH is the impaired absorption of CSF into the venous system, leading to a build-up of CSF and increased ICP [12]. The choroid plexuses predominantly produce CSF, but brain interstitial fluid, ependyma, and capillaries play a poorly defined role in CSF secretion. Absorption of CSF occurs predominantly in the cranial and spinal arachnoid villi, however cranial and spinal nerve sheaths, the cribriform plate, and the adventitia of cerebral arteries contribute as well [13]. CSF movement into the parenchyma drives convective interstitial fluid fluxes within the tissue toward the perivenous spaces surrounding the large deep veins, and later drains into cervical lymphatics. Thus, a problem at those sites may lead to impaired absorption and thus increased CSF levels.

Several conditions interfere with CSF reabsorption, resulting in increased levels of CSF. Dural sinus stenosis can lead to eICP through increased resistance to CSF outflow, venous collapse, and impaired absorption of CSF [14, 15], which in turn leads to the accumulation of catabolites in the brain and CSF [16]. Similarly, Aquaporin-4 (AQP4), the main water channel in astrocytes, facilitates water movement across the cell membrane via polarized channels in astrocytes to help clear catabolites and waste products from the brain [17–19].

The efficient clearance of accumulated waste products relies on astroglial cell-made perivascular tunnels known as the glymphatic system [20]. The cribriform plate, part of the ethmoid bone, serves as a key pathway for glymphatic drainage into nasal turbinates then into lymphatics. This region is critical for connecting intracranial glymphatic flow to the peripheral lymphatic system. This system is increasingly recognized for its role in clearing neurotoxic substances and its implications for neurological diseases such as Alzheimer’s disease and traumatic brain injury.

The glymphatic system may also be actively involved in migraines by influencing cerebral fluid dynamics. For instance, one study highlights increased glymphatic system activity during migraine chronification, which could be linked to altered vascular reactivity characteristic of migraines [21]. This increased activity suggests that the glymphatic system might play a role in managing the neurovascular changes associated with chronic migraine. Further, the glymphatic system has been implicated in the clearance of calcitonin gene-related peptide (CGRP), a key molecule involved in migraine pathology. This suggests that proper function of the glymphatic system could influence the levels of CGRP and thereby impact migraine pathology [22]. A recent a novel computational model suggest that the glymphatic system may be altered during the spreading of depolarization in migraine, in turn altering cerebrospinal fluid flow and bringing up migraine-associated neurological symptoms [23]. Glymphatic system dysfunction resulting from changes in AQP4, impaired glymphatic outflow, and a pro-inflammatory CSF profile may be involved in the development of IIH [24]. These findings suggest various potential roles of the glymphatic system in migraines and states of eICP, though the exact mechanisms and their clinical implications remain subjects of further investigation. As such, understanding these interactions may open new therapeutic avenues for managing and treating migraines and eICP and for differentiating between the two states, highlighting the importance of further research in this area.

### eICP and migraines

eICP is thought to cause headaches by triggering the trigeminovascular system, which consists of trigeminal nerve fibers that innervate pain-sensitive structures in the head, including the dural sinuses [25, 26]. The dural sinuses are richly innervated by trigeminal nerve fibers that contain CGRP, a neuropeptide that is involved in pain transmission. When ICP increases, it can cause congestion of the dural sinuses. This congestion stretches and activates the trigeminal nerve fibers, leading to the release of CGRP and other pain-signaling molecules. The activation of this pathway is thought to be similar to what occurs during a migraine attack, thus contributing to overlapping symptoms between the two conditions.

Although there is no direct correlation between ICP level and headache severity, IIH patients have higher incidence of migraine compared to general population (3,4, 7, 9–12). Additionally, headaches may be resolved temporarily with procedures such as a lumbar puncture that reduce ICP. Additionally, studies have shown that opening pressure, which is a measure of ICP, does not necessarily differ between those with IIH who have headaches and those who do not. Moreover, no significant relationship between opening pressure and headache frequency or severity has been observed. These findings suggest although pressure an important factor, malfunctioned CSF clearance due to eICP causes additional issues which causes the grounds for headache in susceptible individuals.

### Rhinogenic headache

Patients presenting to the otolaryngologist’s office may have symptoms of facial pain/pressure, with subsequent workup of a rhinologic cause, such as CRS. Rhinogenic headache should be considered only if there is an underlying sinonasal pathology. It is common practice to attribute headaches to facial areas—such as the region between the eyes, forehead, or under the eyes. However, in true rhinogenic headache, there must be an active sinonasal condition, such as purulent mucosa or irreversible mucosal changes (e.g., polyps). A simple maxillary sinus cyst, for example, cannot trigger headaches. We believe a true rhinogenic headache is a rare disease and most these patients in fact suffer from eICP and migraine headaches.

Facial pain/pressure is one of the four cardinal symptoms of CRS. These ‘cardinal symptoms’ include 12 weeks with 2 or more of the following: nasal obstruction, thick nasal drainage, facial pain/pressure, and decreased or loss of sense of smell [27, 28]. In addition to these symptoms, there needs to be **confirmation of inflammation** via nasal endoscopy or CT [28]. Even in the presence of visual and radiologic evidence of CRS with or without polyps, the radiographic burden of disease does not correlate with patient-reported pain location and severity [29]. While medical and surgical management can reduce facial pain in the presence of radiologically and endoscopically confirmed CRS [27], patient-reported pain location are not reliable as a means of targeting therapy (i.e., surgically addressing a single sinus) as many patients with disease burden in a single sinus radiographically often report multiple locations of their pain [29].

Facial pain and pressure alone cannot diagnose rhinosinusitis [27, 28]. There is difficulty in establishing a diagnosis when considering that many patients with primary headache disorders also present with autonomic symptoms like rhinorrhea, nasal obstruction/congestion, and facial swelling with triggers such as the weather, seasons, and allergens [28, 30–32]. Thus, when considering the aforementioned ‘cardinal symptoms’ of CRS, the misdiagnosis of CRS in this group of patients with a primary headache disorder is understandable when nasal endoscopy and imaging are not readily available. When confounding symptoms are present, this complete and collaborative evaluation can help avoid diagnostic and treatment delays as well as unnecessary therapies or surgery [33].

Intranasal contact points represent an understudied and controversial area in the CRS and primary headache disorder literature [28, 34]. Some early studies suggested that removing the intranasal contact point surgically resolved or improved headache and facial pain. Many of these studies provided no surgical protocols or follow up periods. Much of the evidence suggesting endoscopic surgical removal of the contact point provides benefit are biased and lack high grade evidence [34].

While CRS typically causes facial pain/pressure through inflammatory mechanisms, similar symptoms can because by eICP through similar mechanisms discussed in the migraine section above, such as trigeminal nerve sensitization. However, facial pain may also be the presenting symptom of an underlying intracranial pathology such as a tumor or vascular pathology that impacts ICP.

### Presentation of eICP in the otolaryngology clinic

Below we present three patient cases that highlight the challenging nature of correct diagnosis and treatment, and the overlapping features between headaches and eICP.

#### Case 1

A 54-year-old woman with a history of sarcoidosis and mild iron-deficiency anemia presented with chronic ear fullness and daily headaches. Initially, she was diagnosed with eustachian tube dysfunction. She was also told that her ear fullness and pain could be related to chronic sinus disease and was advised to use topical nasal steroids and sinus rinses.

Despite daily headaches primarily affecting the areas under her eyes, her forehead, and between her eyes, there was no imaging or clinical evidence of eustachian tube dysfunction or chronic sinusitis. Audiological testing showed normal pure-tone hearing thresholds and a normal tympanogram, while nasal endoscopy revealed only prominent inferior turbinates.

**Fig. 2:**
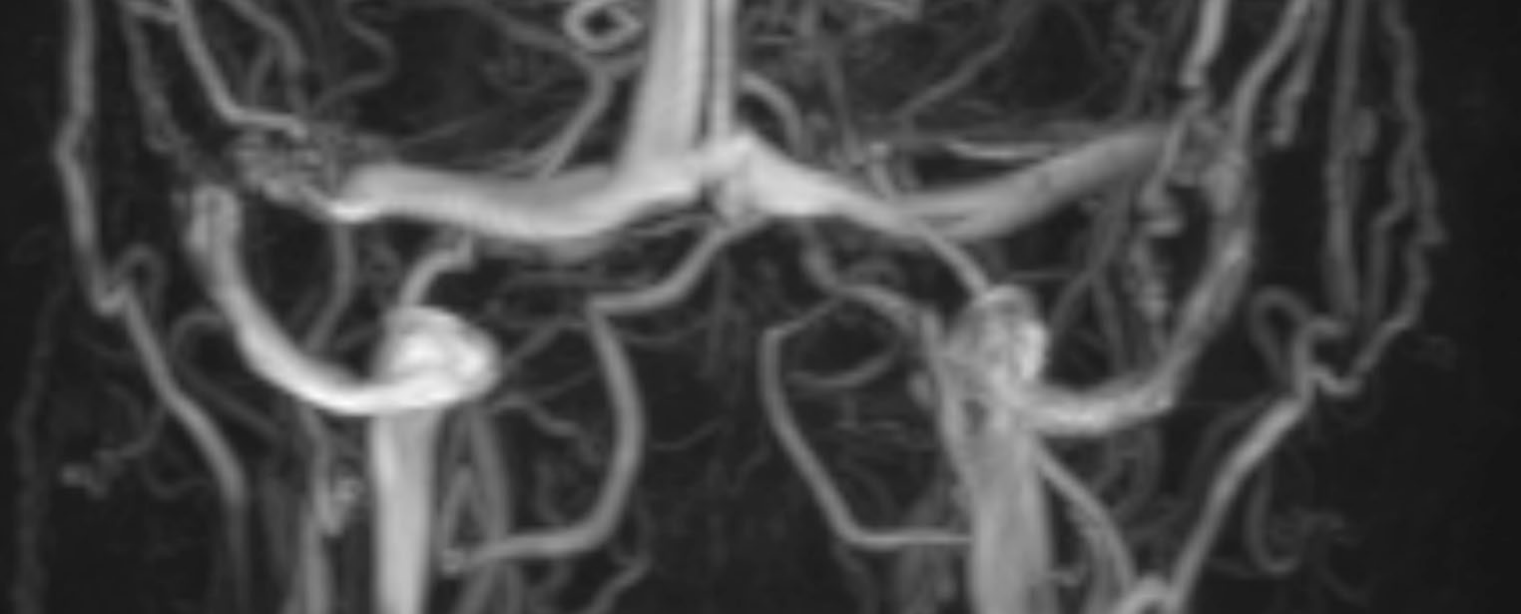
MRV findings of Case 1 demonstrating evidence of a focal right-sided transverse sinus stenosis. Venous manometry is noteworthy for a pressure gradient of 12mmHg across a stenotic segment of the right transverse sinus

Her main symptom was facial pain extending to the area over her ears. Subsequent vertigo and dizziness led her to visit the emergency department, where a stroke workup was negative. However, an MRI revealed lateral transverse sinus stenosis (Fig. 2) and a partially empty sella, with no signs of middle ear or paranasal sinus infection.

Our impression was that her symptoms might be related to eICP. Anemia, even in mild levels, should be taken into consideration seriously as anemia contributes significantly to ICP elevation. We are noticing connective tissue diseases such as sarcoidosis in these patients, we assume these diseases have negative impact on CSF clearances too although medical literature about these is very limited. In this case we tried a short course of acetazolamide combined correction of anemia with a weight loss intervention that successfully resolved her symptoms.

#### Case 2

A 40-year-old woman with borderline diabetes, hypertension, and a body mass index (BMI) of 60 has been experiencing recurrent ear fullness, facial fullness, and nasal congestion for four years. She has a history of middle ear effusion requiring prolonged tympanostomy tube placement. Over this period, she underwent multiple nasal endoscopic examinations, each revealing nonspecific findings—such as serous secretions and mild inferior turbinate hypertrophy—but no evidence of chronic sinusitis (e.g., purulent drainage or irreversible mucosal changes). Following each visit, she was advised to use intranasal steroids and nasal sprays. Due to recurrent facial pain and nasal congestion, she frequently requested oral antibiotics.

On one occasion, when her regular otolaryngologist was unavailable, she was evaluated by a different physician who suggested that her symptoms might fall under the migraine spectrum. A head CT scan revealed no paranasal sinus disease but did show a thinned cribriform ethmoid roof, a tegmen defect, and a partially empty sella—findings highly suspicious for eICP. Notably, the tegmen defect on the same side as the ear tube indicated a possible previous CSF leak.

It can be challenging to convince a patient that her symptoms may be migraine-related, particularly when imaging also points to a long-standing elevation in ICP. In our experience, headaches are the most common complaint among patients with eICP, and these can present clinically as migraines. This raises the question of why CSF pressure is rarely mentioned in migraine pathophysiology (Readers may refer to De Simone and Ranieri [3, 4] for more information).

She was advised to begin taking 50 mg of topiramate, a medication that can help manage migraines and lower ICP, and to follow up with her regular otolaryngologist.

#### Case 3

A 72-year-old male presented with a seven-year history of blurred vision and intermittent diplopia of unknown etiology. Over the past two years, he had progressively worsening balance. He denied vertigo and aural fullness but reported head pressure, bilateral hyperacusis, and non-localizable pulsatile tinnitus. Initial impression was a cerebrovascular episode. Imaging studies (MRI and MRA) showed an empty sella and bilateral optic nerve sheath diameters (ONSD) of 7 mm. A normal optic nerve sheath diameter (ONSD) is generally considered to be around 4–5 mm. Although a neuro-ophthalmologic examination did not confirm papilledema, subsequent MR venography (MRV) revealed complete thrombosis of the right sigmoid sinus and partial thrombosis of the right transverse (lateral) sinus. Further evaluation also showed congenitally narrow left lateral and sigmoid sinuses.

While eICP more commonly affects obese, middle-aged females, it can still occur in older males. In a patient this age, normal pressure hydrocephalus would typically be high on the differential, but imaging findings suggested intracranial changes due to the elevated pressure.

A trial of acetazolamide and other diuretics was attempted but discontinued due to intolerance. The patient was ultimately referred to neurosurgery, where a ventriculoperitoneal (VP) shunt was placed, resulting in immediate symptom relief.

### Diagnostic considerations

Given the overlap in symptoms between eICP and migraines, diagnosing these conditions presents a complex clinical challenge. It is critical to have appropriate workups of patients, which include potential investigations into secondary causes of headaches like IIH, to discern whether the presenting symptoms are primary or secondary disorders and ensure proper treatment is provided.

Several papers outline the workup and evaluation of migraines. The evaluations include primarily a clinical diagnosis as classified by the International Classification of Headache Disorders (ICHD-3), with diagnostic testing reserved for patients with “red flag” signs and symptoms such as thunderclap headache, head trauma, headache onset at age > 50, and papilledema [35, 36]. However, the authors of this paper propose considering the underlying cause of eICP resulting in symptoms that mimic migraines in patients unresponsive to first line migraine medications, especially if they meet the typical at-risk demographic (i.e., young adult, obese, female). Certain clinical findings are used to guide clinicians, with one finding most associated with IIH being papilledema. However, it is important to note that though papilledema may be seen in IIH, many cases of migraines have been reported in patients with IIHWOP [37, 38]. **Thus**,** a normal fundoscopic exam insufficient for ruling out IIH or meICP as the underlying cause of a patient’s presentation.** Diagnostic imaging findings that can serve as helpful tools and indicators for eICP underlying a patient’s headache, with three key findings described below.

Empty sella syndrome has been reported in previous studies as a feature of IIH, and is seen in the cases presented above. Empty sella results from CSF filling the sella turcica, and in IIH results from intrasellar herniation of arachnoid membrane and CSF through the diaphragm sella [39, 40]. Maralani et al. demonstrated that among 43 patients and 43 controls assessed for MRI findings of IIH, a partially empty sella described as a pituitary gland occupying < 50% of the pituitary fossa had a specificity of 95.3% for IIH [41]. Additionally, one study found that empty sella in IIH is largely reversible with correction of the IIH particularly in patients with a more acute presentation [39], and another study found that the absolute pituitary area was significantly increased by approximately 18% in patients with IIH following treatment compared with pre-treatment [42]. Therefore, the finding of empty or partially empty sella can increase certainty of underlying IIH, and may be used to monitor treatment efficacy in some cases.

The second imaging correlate described in previous studies and seen in the cases outlined above is increased ONSD, often described as a distension > 2 mm [40]. A study by Agid et al. comparing MRI and MRV findings in 29 patients with proven IIH and 56 control subjects found that 66.7% of study patients showed ONSD, compared with 17.9% of controls [43]. Bidot et al. demonstrated that patients with IIH had a larger ONSD as compared with controls [44] and a study by Jeub et al. found that increased ONSD predicted IIH with an 81% sensitivity and 80% specificity [45], again indicating utility as a diagnostic tool for IIH. Jeub et al. demonstrated that 24 h following lumbar puncture, ONSD decreased significantly, with a mean reduction of 0.4 mm in the right eye and 0.5 mm in the left eye [45]. Similarly, a study by Wang et al. highlighted that ONSD measurements correlate with ICP levels, and that ONSD decreased significantly with correction of eICP [46]. Like empty sella, ONSD is a non-invasive method of monitoring disease progression and response to treatment in IIH.

One of the most well-described imaging correlates is dural sinus stenosis as presented case 1. Several studies have elucidated a notable association between dural sinus stenosis and IIH, suggesting a potential causative role. Among the most sensitive and specific markers for IIH is bilateral sinus stenosis > 50%, which is present in nearly 100% of people with IIH [47, 48]. Overall, these imaging findings serve as valuable adjuncts in assessing and managing ICP and can aid in clinical decision-making when approaching headache diagnosis and management, with further investigation being pursued in the presence of these imaging abnormalities.

### Considerations in management

Frequently, we will see eICP in the clinic; as a cause for migraines is identified, several approaches to management can be effective. The challenges in diagnosis and treatment warrant a multidisciplinary approach to treatment that addresses both conditions. The treatment goals are twofold: improving quality of life through symptom management and treating the underlying cause of the eICP. Among the least invasive options is lifestyle modifications that include stress reduction techniques, diet modification, and weight loss, particularly in overweight or obese individuals [49]. Medications targeting migraine symptoms such as acetaminophen, nonsteroidal anti-inflammatory drugs, triptans, antiemetics, and recently Gepants may be beneficial in addressing migraine symptoms also in patients who have eICP [49]. Migraine prophylactic medications like beta-blockers, acetazolamide, and anticonvulsants can reduce headaches’ frequency [50]. However, surgical interventions, such as VP shunts and transverse sinus stenting, may be used in emergent situations [50]. Identifying and addressing other risk factors for eICP, including systemic conditions such as anemia, sarcoidosis, and chronic kidney disease, and medications such as steroids is also an important aspect of the control of eICP and associated symptoms. Consequently, careful screening of patients for OSA may be integral in ultimately the symptoms associated with eICP.

## Discussion

Patients presenting with CRS-like or migraine symptoms may exhibit signs suggestive of eICP such as ear fullness, muffled hearing, and tinnitus, which do not resolve with conventional CRS or migraine treatments. The similarities in symptoms between migraines and eICP underscore the necessity for careful differential diagnosis.

### Other factors that may contribute to headaches in the setting of eICP include

- **Individual variability in pain sensitivity**: Some individuals may be more sensitive to pain signals from the trigeminal system.
- **Underlying migraine predisposition**: Individuals with a history of migraine may be more likely to experience headaches in the setting of IIH.
- **Venous sinus stenosis**: Narrowing of the venous sinuses, regardless of whether it is a cause or consequence of eICP, may contribute to headache.
- **Changes in cerebral blood flow**: eICP may disrupt the normal regulation of cerebral blood flow.

However, eICP is not the only factor involved in headaches. Any true inflammatory condition in any part of the body can trigger migraine in susceptible patients. These includes CRS patients but also other conditions like gastrointestinal or respiratory infections, autoimmune diseases including Hashimoto’s Thyroiditis, Sjogren’s syndrome, Celiac disease, multiple sclerosis, and many more.

The overlap in clinical presentations among patients with CRS, migraines, and eICP poses a diagnostic challenge. Otolaryngologists and neurologists must collaborate closely to ensure accurate diagnoses and appropriate management. Enhanced awareness and understanding of the broader spectrum of symptoms associated with eICP can prevent misdiagnosis and promote better patient outcomes.

It is crucial to recognize the potential for eICP in patients presenting with CRS or migraine-like symptoms. This understanding can guide more effective management strategies and prevent misdiagnoses, leading to improved patient care in otolaryngology and neurology.

### Addendum: quick review of CSF physiology and pathophysiology

#### CSF production

Involves blood filtration through the choroid plexus and active transport of ions, with CSF secretion regulated by factors such as blood pressure and composition.

#### CSF circulation and Re-absorption

CSF flows through the ventricular system and is reabsorbed at the arachnoid villi, facilitated by endothelial and epithelial transport. CSF is also absorbed along the cranial and spinal nerves.

#### Glymphatic pathway

Indicates the role of lymphatic vessels in CSF absorption, linking CNS fluid balance with the lymphatic system.

#### Monro-kellie doctrine

Highlights the importance of volume equilibrium within the cranial compartment for maintaining normal intracranial pressure. Although we didn’t elaborate about this doctrine, it is an important concept that all readers should be familiar with.

#### Pathophysiology of increased intracranial pressure

Discusses the consequences of eICP due to various pathologies affecting brain tissue, blood, or CSF volume.
